# Supplementary material for: The causal role of circulating inflammatory markers in osteoporosis: a bidirectional Mendelian randomized study
Source: Front Immunol. 2024 Jul 18;15:1412298. doi: 10.3389/fimmu.2024.1412298 (PMC11291241; doi:10.3389/fimmu.2024.1412298)
Supplement: Supplementary file 1 [file DataSheet_1.zip › supplementary material/OSTEOPOROSIS-CIM/GCST90274765.tsv.gz_forest.pdf]

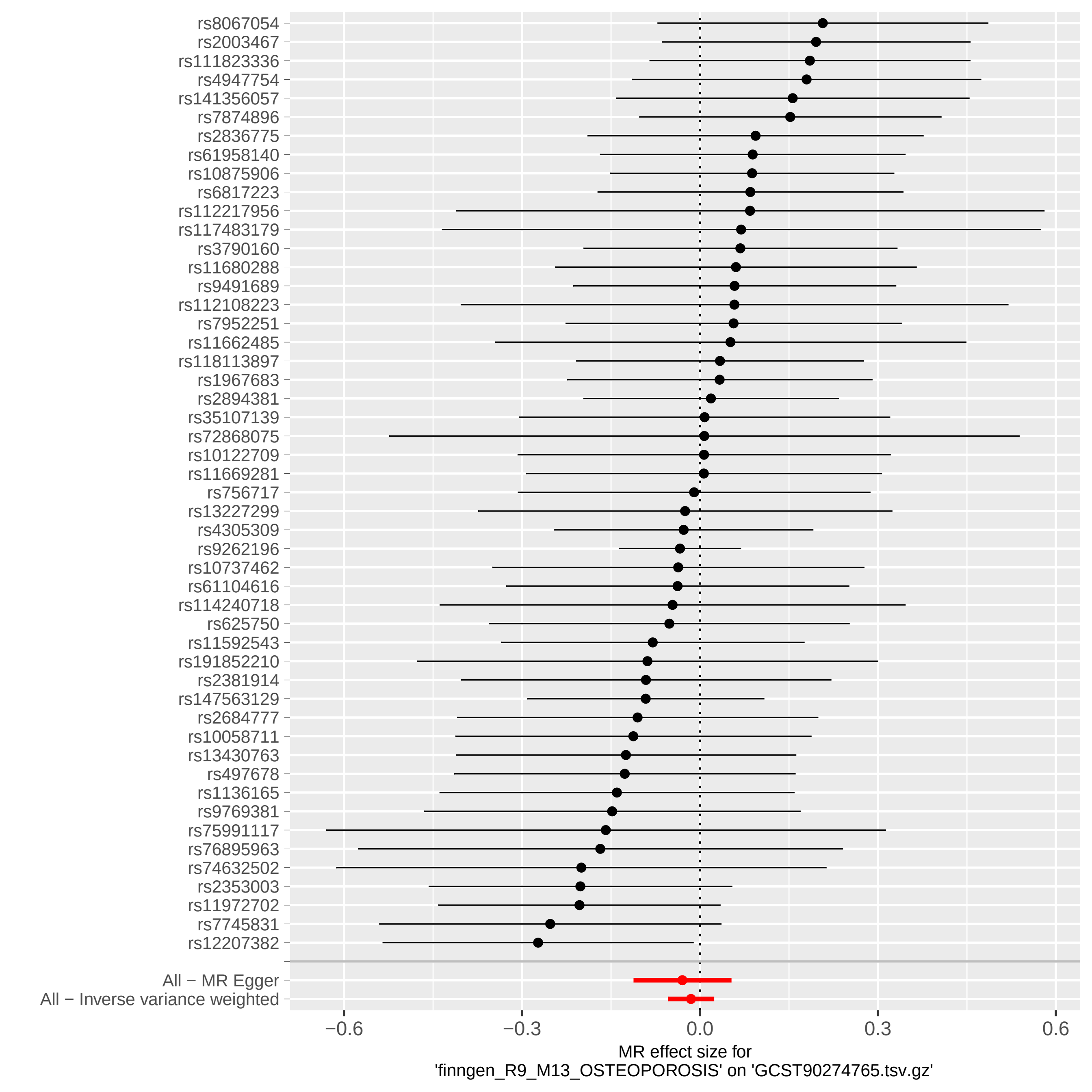
rs8067054
rs2003467
rs111823336
rs4947754
rs141356057
rs7874896
rs2836775
rs61958140
rs10875906
rs6817223
rs112217956
rs117483179
rs3790160
rs11680288
rs9491689
rs112108223
rs7952251
rs11662485
rs118113897
rs1967683
rs2894381
rs35107139
rs72868075
rs10122709
rs11669281
rs756717
rs13227299
rs4305309
rs9262196
rs10737462
rs61104616
rs114240718
rs625750
rs11592543
rs191852210
rs2381914
rs147563129
rs2684777
rs10058711
rs13430763
rs497678
rs1136165
rs9769381
rs75991117
rs76895963
rs74632502
rs2353003
rs11972702
rs7745831
rs12207382
All − MR Egger
All − Inverse variance weighted
−0.6
−0.3
0.0
0.3
0.6
MR effect size for
'finngen_R9_M13_OSTEOPOROSIS' on 'GCST90274765.tsv.gz'
